# Differential Effects of Educational and Occupational Cognitive Reserve on Foreign-Accented Speech Evaluation by Older Adults

**DOI:** 10.3390/brainsci16030280

**Published:** 2026-02-28

**Authors:** Jolanta Sypiańska, Zuzanna Cal

**Affiliations:** 1 Department of Linguistics, Faculty of Humanities, Kampus Piastów, University of Szczecin, 70-453 Szczecin, Poland; 2 Department of Contemporary English and Multilingualism, School of Languages and Literatures, Faculty of English, Adam Mickiewicz University, 60-780 Poznań, Poland; zuzanna.cal@amu.edu.pl

**Keywords:** cognitive reserve, foreign accent rating, late adulthood, older adult listeners

## Abstract

**Highlights:**

**What are the main findings?**

- Composite cognitive reserve showed no reliable association with adult listeners’ ratings of foreign-accented speech, apart from a marginal effect on perceived accent strength.
- Occupational and educational components of cognitive reserve exhibited robust but opposing effects on comprehensibility, perceived intelligibility and listening effort.

**What are the implications of the main findings?**

- Cognitive reserve is not a unitary construct in speech perception and should be examined through its distinct experiential components.
- Lifelong educational cognitive engagement may support more efficient processing of acoustically variable speech in later adulthood.

**Abstract:**

**Background/Objectives**: Older adults are increasingly exposed to foreign-accented speech in everyday communication, yet such speech is known to increase perceptual and cognitive demands even when intelligibility is preserved. While previous research has documented age-related differences in processing foreign-accented speech, less is known about how individual differences in cognitive reserve shape older adults’ subjective evaluations of accented speech. The present study examined whether cognitive reserve is associated with senior listeners’ ratings of foreign-accented English across multiple perceptual dimensions. **Methods**: Thirty native English-speaking British adults aged 66–84 years completed an online foreign accent rating task. Participants rated non-native English speech on four dimensions: perceived accent strength, comprehensibility, perceived intelligibility and listening effort. Cognitive reserve was operationalized using a multidimensional proxy approach incorporating educational attainment, occupational complexity and leisure activities. These components were combined into a composite cognitive reserve score and also examined separately. The ratings were analyzed using cumulative link mixed models with the participant as a random intercept. Self-reported experience with foreign-accented speech was included as a covariate in all models. **Results**: Composite cognitive reserve showed no significant association with comprehensibility, perceived intelligibility, or listening effort and only a marginal negative association with perceived foreign-accent strength. When cognitive reserve was decomposed into its components, educational cognitive reserve significantly predicted higher comprehensibility and perceived intelligibility ratings and higher listening effort ratings. Occupational cognitive reserve showed significant effects in the opposite direction for comprehensibility, perceived intelligibility and listening effort; however, it was also associated with lower perceived accent strength. Self-reported experience with foreign-accented speech was not a significant predictor for any outcome. **Conclusions**: These findings indicate that cognitive reserve does not exert a uniform influence on older adults’ perception of foreign-accented speech. Instead, occupational and educational components of cognitive reserve show distinct and opposing associations with perceptual evaluations. The results highlight the importance of using multidimensional approaches to cognitive reserve when examining individual differences in speech perception in later adulthood.

## 1. Introduction

In increasingly multilingual societies, older adults are frequently exposed to foreign-accented speech in everyday communication, which includes healthcare encounters, social services and mass media. Foreign-accented speech differs systematically from native speech at the phonetic, segmental and prosodic levels as it is potentially rich in cross-linguistic influences from a speaker’s background languages to foreign language production [1,2], slower speech and/or articulation rate and various types of disfluencies. The increased variability in the acoustic signal introduces perceptual uncertainty and has been shown to slow processing and increase cognitive demands for listeners, even when overall intelligibility remains relatively high [3,4,5]. A substantial body of behavioral research demonstrates that listeners can adapt to foreign-accented speech with exposure, leading to improved recognition accuracy and processing efficiency [6,7]. However, the majority of this evidence derives from studies with younger adults. Research focusing on older listeners suggests a more complex pattern. While older adults often achieve high levels of comprehension accuracy when listening to foreign-accented speech, they typically experience slower processing, increased difficulty and reduced adaptation relative to younger adults [8]. When studying subjective speech perception, it is crucial to distinguish between accentedness, comprehensibility and intelligibility. Following Munro and Derwing [9], intelligibility refers to the extent to which a given utterance is actually understood by a listener, typically measured by scoring listeners’ transcriptions of speech [10]. In the present study, however, intelligibility was assessed via listeners’ self-reported ratings of how much they believed they understood. We therefore operationalize intelligibility as perceived intelligibility rather than objective transcription-based accuracy. In contrast, comprehensibility reflects a listener’s perception of how easily an utterance is understood, while accentedness reflects the degree to which a speaker’s pronunciation deviates from native norms [11,12]. Importantly, L2 speakers can be highly comprehensible despite strong accents, illustrating that accentedness does not necessarily impede functional understanding [13,14,15]. Comprehensibility can be influenced not only by phonetic accuracy but also by factors such as prosody, fluency and speaking rate, with faster-than-average L2 speech often perceived as easier to understand [16]. While comprehensibility captures listeners’ perceived processing ease, perceived intelligibility reflects their subjective judgment of comprehension success (i.e., how much of the message they believe they understood). Although intelligibility has been traditionally operationalized in terms of objective comprehension accuracy (e.g., transcription performance), more recent work in speech perception distinguishes between objective intelligibility and perceived understanding. For example, subjective intelligibility judgments can deviate from objective speech recognition outcomes, particularly in older listeners, suggesting that listeners’ beliefs about how much they understood are influenced by factors beyond raw decoding accuracy [17,18]. Similarly, experimental studies comparing objective and subjective intelligibility measures demonstrate that subjective listener judgments capture aspects of perceptual experience not fully reflected in objective scoring [17]. In addition, research on metacognitive monitoring in aging indicates that self-monitoring of comprehension is itself a cognitive process that varies with age and educational experience [19]. These findings support treating perceived intelligibility as a metacognitive judgment of comprehension outcome, conceptually distinct from both objective intelligibility and perceived ease of processing (comprehensibility).

Importantly, cognitive reserve is increasingly conceptualized as a multidimensional construct in which different life-course experiences contribute through partially distinct neurocognitive mechanisms rather than functioning as interchangeable indicators of a single latent factor [20,21]. Contemporary models distinguish between neural efficiency, capacity, flexibility and compensatory recruitment, emphasizing that reserve expression depends on task demands [22]. Within this framework, educational attainment is often treated as a relatively stable early-life proxy reflecting formal learning and crystallized knowledge, whereas occupational complexity captures sustained cognitive engagement across adulthood [23,24]. Longitudinal evidence indicates that higher occupational complexity is associated with better executive functioning and slower cognitive decline in aging [25] and sustained cognitive engagement has been proposed to strengthen adaptive control systems [26]. Executive control mechanisms, including working memory, attentional allocation and cognitive flexibility, are recruited during effortful speech perception, particularly under conditions of acoustic degradation or variability [27,28]. Processing foreign-accented speech increases demands on such top-down control processes to support lexical disambiguation and adaptation to phonetic variability. In contrast, leisure activities, which include reading, hobbies, games and social engagement, are thought to support cognitive reserve through broader mechanisms such as general mental stimulation, attentional engagement and social-cognitive enrichment [26,29]. While these experiences may promote overall cognitive vitality and maintain processing efficiency, their effects on executive control networks may be less direct or domain-specific than those associated with occupational complexity. Accordingly, in perceptually challenging listening contexts, leisure-related reserve might support adaptation to novel or degraded speech more diffusely, facilitating general attentional alertness or sustained engagement, but not necessarily enhancing the fine-grained executive functions required for rapid lexical disambiguation. Within reserve frameworks that emphasize efficiency and flexibility [22,29], it is therefore theoretically plausible that educational supports accurate lexical access and general language comprehension; occupational may facilitate processing efficiency and adaptive control in perceptually demanding contexts; and leisure-based reserve may contribute to global listening resilience and sustained attentional engagement. This tripartite distinction allows for testable hypotheses in which educational, occupational and leisure experiences differentially predict subjective speech evaluations, perceptual adaptation and the recruitment of specific executive control mechanisms during effortful listening.

This framework provides a useful theoretical lens for understanding why older adults may successfully comprehend foreign-accented speech while simultaneously experiencing elevated processing demands. Importantly, these age-related differences cannot be fully attributed to peripheral hearing loss. Studies controlling for hearing acuity have reported mixed findings ranging from minimal age-related effects [25,26] to persistent disadvantages for older listeners [27,28]. These discrepancies point to an important contribution of cognitive factors beyond auditory sensitivity. Neurocognitive evidence further supports the view that foreign-accented speech places particular demands on older adults during online language processing. Using event-related potentials (ERPs), Abdollahi et al. [11] showed that although older adults achieved high comprehension accuracy for both native- and foreign-accented sentences, their neural responses differed markedly as a function of accent. Semantic violations elicited a robust N400 effect in native-accented sentences, whereas no corresponding N400 effect was observed for foreign-accented sentences. This absence suggests less efficient or weaker activation of lexical–semantic representations when processing non-native phonetic input, even when comprehension is ultimately successful. In contrast, grammatical violations elicited comparable P600 responses across accent types, indicating relative preservation of syntactic reanalysis mechanisms. The pattern aligns with ERP findings in younger adults, which show that foreign-accented speech does not block semantic access but delays or attenuates N400 responses that are symptomatic of increased processing demands [30,31,32]. Crucially, Abdollahi et al. [11] demonstrated that such disruptions to early semantic processing are more pronounced in older adults, which points to a dissociation between behavioral comprehension outcomes and underlying processing effort in aging.

Despite these advances, most prior work has focused on sentence-level accuracy or neurophysiological indices of processing. Far less is known about how older adults explicitly evaluate foreign-accented speech across perceptual dimensions that are central to everyday communication, such as perceived accent strength, comprehensibility, perceived intelligibility and listening effort. This is a critical gap, as accuracy-based measures alone may obscure substantial differences in subjective processing demands. Indeed, older adults may successfully understand accented speech while simultaneously experiencing elevated cognitive effort, a factor with important implications for communication fatigue, participation and well-being. Another unresolved issue concerns the pronounced inter-individual variability observed among older listeners. Some older adults cope efficiently with foreign-accented speech, whereas others experience marked difficulty under comparable conditions. A prominent framework for explaining such variability is cognitive reserve (CR), defined as the capacity to maintain cognitive performance despite age-related neural changes or neuropathology [33,34]. Cognitive reserve is thought to reflect lifelong engagement in intellectually stimulating activities, education, occupational complexity and enriched environments. Importantly, recent research has emphasized that cognitive reserve cannot be adequately captured by single, static proxies such as years of formal education alone. Neuropathological evidence demonstrates that dynamic measures of cognitive reserve, such as vocabulary knowledge or intellectual functioning, more effectively predict cognitive performance in the presence of Alzheimer’s disease pathology than education alone [27]. These findings suggest that cognitive reserve continues to develop across the lifespan and that dynamic proxies better capture ongoing enrichment beyond early-life schooling. Evidence from healthy older adults further supports this multidimensional view. Using the Cognitive Reserve Questionnaire (CRQ), Grasso et al. [28] showed that cognitive reserve is positively associated with executive functions, verbal fluency and global cognitive performance in older adults without cognitive impairment. While years of education significantly contributed to reserve levels, additional experiential factors, including occupational complexity, multilingualism, reading habits and engagement in cognitively stimulating activities, also played a substantial role. These results underscore that cognitive reserve reflects the cumulative impact of diverse life experiences rather than education alone. Multidimensional approaches to cognitive reserve have been shown to provide greater explanatory power for age-related cognitive variability. For example, Grotz et al. [35] demonstrated that a weighted index combining education, occupational attainment and leisure activities more effectively moderated cognitive decline than standard proxy measures. All in all, these findings highlight the importance of adopting composite, experience-based measures when examining how cognitive reserve supports performance under challenging perceptual conditions. Age-related changes in neural plasticity and auditory processing further constrain speech perception in older adults. Cognitive decline, reductions in white matter integrity, and decreased dopaminergic signaling are associated with slower learning and less efficient compensatory networks [36]. Behavioral studies indicate that older adults benefit from experience-based training on challenging perceptual tasks, such as identification of non-native phonetic contrasts, but show limited gains in pre-attentive discrimination or neural measures like mismatch negativity (MMN) [36]. These findings suggest that training may enhance attentive, conscious processing of non-native contrasts, whereas pre-attentive mechanisms remain relatively inflexible in aging, highlighting the role of experience and cognitive reserve in shaping perceptual learning outcomes. Applied to foreign-accented speech perception, this framework suggests that higher cognitive reserve may enable older adults to deploy more flexible and efficient processing strategies when faced with increased phonetic variability. Such reserve-related mechanisms may not necessarily enhance objective intelligibility but may reduce perceived listening effort and increase comprehensibility, even when accent strength remains unchanged.

The present study builds on prior behavioral, neurocognitive and training research by examining older adults’ perceptual ratings of foreign-accented speech across accent strength, comprehensibility, perceived intelligibility and listening effort. Crucially, we investigate whether individual differences in cognitive reserve, assessed using a multidimensional proxy approach, account for variability in these perceptual evaluations. This study aims to clarify how listener-related cognitive and experiential factors shape subjective speech perception in aging societies. In practice, the aim of the present study is to examine the relationship between senior listeners’ cognitive reserve and their perceptual evaluation of non-native English speech. In particular, this study investigated whether individual differences in cognitive reserve are associated with variability in the perception of foreign-accented speech across multiple perceptual dimensions, including perceived degree of foreign accent, comprehensibility, perceived intelligibility and listening effort. By combining detailed lifetime measures of education, occupational complexity and leisure activities with a controlled foreign accent rating task, this study seeks to clarify how cognitive reserve contributes to foreign accent perception in later adulthood. This study addressed the following research questions:

RQ1. Is cognitive reserve associated with senior listeners’ ratings of foreign-accented speech?
RQ2. Do different components of cognitive reserve (education, occupational history and leisure activities) differentially predict foreign accent ratings?
RQ3. Is cognitive reserve more strongly associated with effort-related dimensions than with accent strength?

Based on prior behavioral, neurocognitive and cognitive reserve research in the literature [7,11,22,24,26], this study tested the following hypotheses:

**H1.**  *Higher overall cognitive reserve will be associated with more favorable perceptual evaluations of foreign-accented speech in older adults, reflected in lower perceived accent strength, higher comprehensibility and intelligibility and reduced listening effort*.

**H2.**  *Different components of cognitive reserve will differentially predict perceptual ratings of foreign-accented speech, such that occupational cognitive reserve will be more strongly associated with improved comprehensibility, perceived intelligibility and reduced listening effort than educational cognitive reserve, while leisure-based cognitive reserve is expected to contribute more modestly, supporting general engagement and attentional persistence during listening*.

**H3.**  *Cognitive reserve will be more strongly associated with effort-related dimensions of foreign-accented speech perception (listening effort) than with accent strength, underscoring compensatory processing mechanisms rather than changes in perceptual categorization with aging*.

## 2. Materials and Methods

### 2.1. Participants

The participants completed a background questionnaire which aimed at collecting demographic, linguistic, socioeconomic, health and lifestyle metadata. The demographic information included date of birth (from which age in years was computed), place of birth (city and country) and sex at birth. Information on nationality and migration history was collected, including country or countries of residence across adulthood and age at migration when applicable. Language background data included native language(s), other languages spoken fluently and daily use of non-native languages. Social and household characteristics were assessed through current marital status (single, partnered, widowed, divorced) and living situation (alone, with partner, with family, other). Residential context was documented by type of residence (urban, suburban or rural) and current housing arrangement (private home, assisted living or institutional setting). Socioeconomic status was evaluated using self-reported perceived financial comfort (very difficult, difficult, adequate, comfortable). Retirement-related information included current employment status (retired, semi-retired or still working), age at retirement and primary reason for retirement (health, age, other). Medical history was assessed using yes/no questions covering major neurological, psychiatric and systemic conditions, which included dementia or mild cognitive impairment, stroke or cerebrovascular disease, traumatic brain injury requiring hospitalization, Parkinson’s disease, epilepsy, psychiatric disorders, cancer (with type and year specified), diabetes, hypertension and cardiovascular disease. The participants also reported current medications, including the use of psychotropic drugs. Sensory and functional status was evaluated through self-reported uncorrected vision and hearing impairments as well as mobility limitations. Finally, lifestyle-related metadata covered smoking status (never, former, current) and alcohol consumption (none, occasional, regular).

The participants were recruited online via the Prolific platform and received GBP 4.50 for completing this study, in line with Prolific’s recommended payment guidelines. Prior to completing any tasks, participants were provided with an information sheet detailing this study’s aims, procedures, estimated duration and ethical considerations. They provided informed consent after reading this information and could withdraw at any time without penalty.

The sample consisted of 30 senior adults (17 females, 13 males) aged 66–84 years (M = 69.3, SD ≈ 4.6). All participants were British nationals and had spent the majority of their adulthood in the United Kingdom. All were born in the UK (England, Wales or Scotland). Two participants reported periods of residence outside the UK (South Africa and Australia), although these did not constitute the majority of their adult lives. The participants were generally healthy, community-dwelling older adults. Over half of the sample reported no major medical conditions (53.3%). The most commonly reported diagnoses were hypertension (30.0%), diabetes (10.0%) and cardiovascular disease (10.0%), and two participants reported a history of cancer. No participant reported current use of psychotropic medication in the structured questionnaire; however, a small number listed antidepressant or anxiolytic agents in the free-text medication field. Most participants reported no sensory or functional impairments (80.0%), with a small number reporting uncorrected vision or hearing difficulties or mobility limitations. With respect to lifestyle factors, the majority had never smoked (63.3%) or were former smokers (33.3%), and alcohol consumption was predominantly occasional (53.3%) or regular (26.7%), with no reports of heavy use. All participants were native speakers of English. Four participants reported speaking an additional language fluently (French, Indonesian, Afrikaans or Welsh), while the remaining participants reported no additional fluent languages. Only one participant reported daily use of a non-native language. All others indicated no regular daily use of languages other than English. The majority of participants were married or partnered (*n* = 22, 73.3%). Four participants were widowed (13.3%), three were divorced (10.0%) and one participant reported being single (3.3%). Most participants lived in suburban (*n* = 16, 53.3%) or rural areas (*n* = 10, 33.3%), with a smaller proportion residing in urban settings (*n* = 4, 13.3%). All participants reported living in a private home. Regarding perceived financial comfort, the majority described their situation as adequate (*n* = 17, 56.7%) or comfortable (*n* = 10, 33.3%), while a small number reported financial difficulty (*n* = 3, 10.0%). Most participants were retired (*n* = 26, 86.7%), with a smaller proportion being semi-retired (*n* = 3, 10.0%), and one participant still in employment (*n* = 1, 3.3%). Among retired and semi-retired participants (*n* = 29), retirement was most commonly attributed to age-related reasons (*n* = 22, 75.9%), followed by other reasons (*n* = 4, 13.8%) and health-related reasons (*n* = 3, 10.3%). The mean age at retirement was 63.5 years (range: 55–70).

### 2.2. Procedure

From the participant’s perspective, this study unfolded in the following sequence. After accessing the online study link, participants were first presented with an information sheet describing the study’s purpose, estimated duration, tasks, ethical considerations, and their rights as participants, including the right to withdraw at any time without penalty. Participants provided informed consent before proceeding. Next, participants completed a background questionnaire collecting demographic, linguistic, socioeconomic, health, and lifestyle information. This was followed by the cognitive reserve questionnaire, covering education, occupational history and engagement in leisure activities across the lifespan. Finally, participants completed the foreign-accent rating task. For each recording, participants rated four perceptual dimensions, degree of foreign accent, comprehensibility, perceived intelligibility and listening effort, on separate 0–10 Likert-type scales. The entire study was conducted online via Qualtrics and took approximately 25 min to complete.

### 2.3. Cognitive Reserve Assessment

Cognitive reserve was operationalized using a lifestyle questionnaire covering three main domains: educational attainment, occupational path and leisure activities. These domains were selected in line with established approaches to estimating cognitive reserve based on lifetime exposure to cognitively stimulating experiences. First of all, educational attainment was assessed through multiple complementary measures. The participants reported their highest completed educational level for which an official diploma or certificate was obtained, ranging from no diploma or primary education to doctoral-level degrees. In addition, participants were asked to list all educational diplomas or degrees obtained and to report the total number of years of formal education completed across primary, secondary and higher education. The participants also indicated whether they had completed any professional or vocational training courses lasting at least six months, excluding formal degree programs and reported the number and nature of such courses when applicable. Educational attainment was categorized into five levels adapted to the British education system: no formal qualification or primary education only (up to six years of schooling); lower secondary education, such as General Certificate of Secondary Education (GCSE) or equivalent qualifications (approximately seven to eleven years of schooling); upper secondary education, including A-levels, Scottish Highers, or equivalent vocational qualifications (approximately twelve to thirteen years of schooling); post-secondary non-university education such as further education, higher national certificates or diplomas and foundation degrees (approximately fourteen to sixteen years of schooling); and university education, encompassing undergraduate and postgraduate degrees (Bachelor’s, Master’s, and Doctoral degrees), corresponding to seventeen or more years of schooling. In addition to this categorical variable, total years of education (continuous) and number of long-term training courses (count) were included as indicators of educational exposure.

Second of all, occupational history was assessed using a retrospective questionnaire focusing on overall employment exposure and occupational complexity. This study participants first reported the total number of years spent in paid employment across their lifetime, excluding periods of career interruption. Current working time regime (full-time, part-time or not working) was recorded to capture present occupational engagement. The participants then provided information about their main occupational status prior to retirement (employed, self-employed, unemployed or in another work-related situation). They reported their most recent or pre-retirement job title and, when possible, indicated the corresponding International Standard Classification of Occupations (ISCO-08) category [37]. ISCO categories were used to classify occupations according to skill level and task complexity. The participants who were unfamiliar with ISCO classifications could select an “I do not know” option, with occupational coding performed subsequently by the researchers when sufficient information was available. Career interruptions were assessed by asking participants whether they had experienced career breaks during their employment and, if so, to indicate the approximate cumulative duration of these interruptions. From this section, key occupational variables were derived for analysis, including total years of paid employment, occupational skill level of the last occupation and cumulative exposure to career interruptions. These measures were used as indicators of occupational contribution to cognitive reserve.

Lastly, engagement in leisure activities was assessed retrospectively across five life stages: childhood and adolescence (≤17 years), early adulthood (18–29 years), adulthood (30–49 years), late adulthood (50–65 years) and older adulthood (≥66 years). For each life stage, participants indicated which leisure activities they practiced from a predefined list encompassing physical, social, intellectual and cultural domains. Physical activities included sports, walking or hiking and dancing. Social activities comprised meeting friends or family, participation in charitable activities or volunteering, participation in clubs or associations and other regular social engagements. Intellectual activities included reading books and newspapers or magazines, completing crosswords or puzzles, playing strategy games, and using new technologies. Cultural activities included attending the theatre, concerts or cinema, visiting museums or exhibitions, travelling and engaging in artistic activities such as painting, drawing, or music. Participants could also indicate that none of the listed activities were practiced during a given life stage. For analytical purposes, participation in leisure activities was examined both by life stage and cumulatively across the lifespan, allowing the derivation of indices reflecting long-term engagement in cognitively, socially and physically stimulating activities. These indices were used as proxies of lifestyle-related cognitive reserve.

To combine the three domains into a single composite cognitive reserve (CR) score, each domain-specific score (CR_Education, CR_Occupation, CR_Leisure) was first standardized using a z-score transformation. After z-scoring, the three standardized scores were averaged to produce the final composite CR score for each participant. To facilitate interpretation and comparability, the resulting composite scores were then linearly scaled to a 0–100 range with higher values reflecting greater overall cognitive reserve (CR_composite).

To quantify the education component of cognitive reserve, we derived a composite score reflecting three aspects of educational experience: the highest level of formal education attained, the total years of formal education, and continued educational engagement beyond the highest qualification. The highest educational qualification was coded on an ordinal scale ranging from 1 (no diploma or primary education) to 6 (doctoral degree), reflecting increasing potential contribution to cognitive reserve. Total years of formal education, including primary, secondary and higher education, were treated as a continuous variable and subsequently standardized (z-score) to ensure comparability with other indicators. Continued educational engagement was captured by counting additional diplomas or degrees beyond the highest qualification and professional or vocational training courses lasting at least six months. This engagement measure was also standardized. The three standardized indicators, including the highest education level, years of education and educational engagement, were then averaged to produce a single education composite score for each participant.

The occupational component of cognitive reserve was derived from each participant’s lifetime employment history, taking into account the total duration of paid work, the complexity of their main occupation, and the occurrence of career breaks. Firstly, the total number of years spent in paid employment across the lifespan was recorded for each participant, excluding career breaks. If a participant reported career breaks, the total duration of these interruptions was subtracted from their reported years of employment to obtain the adjusted years of employment. For cases where the exact duration of career breaks was not specified but indicated as “Yes, 4 years or more,” a fixed value of 4 years was used. Next, participants’ main occupational status prior to retirement was categorized according to the ISCO-08 classification or, when not available, according to the reported job title. Occupations were grouped into three levels of cognitive complexity: high (managers and professionals), medium (technicians and associate professionals), and low (clerical, service, or other roles). Finally, the adjusted years of employment were multiplied by a weighting factor reflecting occupational complexity to generate a weighted occupational score: (1) high-complexity occupations weighted by 1.2; (2) medium-complexity occupations weighted by 1.0; (3) low-complexity occupations weighted by 0.8. The resulting weighted occupational score combines both the duration and cognitive demands of participants’ work histories and serves as one component of the overall cognitive reserve composite score.

The participants reported the leisure activities they engaged in across five life periods: childhood and adolescence (≤17 years), early adulthood (18–29 years), adulthood (30–49 years), late adulthood (50–65 years), and older adulthood (≥66 years). For each period, activities were categorized into types such as physical activities (e.g., sports, walking, hiking, dancing), cognitive activities (e.g., reading, puzzles, brain games, strategy games), social activities (e.g., meeting friends or family, clubs, volunteering), cultural activities (e.g., theater, concerts, museums, exhibitions, artistic activities) and use of technology (e.g., computer, tablet, smartphone). For each participant, the number of unique activity types engaged in per age period was counted. To account for the continued contribution of leisure activities to cognitive reserve across the lifespan, counts from adulthood and late adulthood (ages 30+) were weighted slightly higher than those from childhood and early adulthood. Specifically, activities reported for adulthood, late adulthood and older adulthood were multiplied by a factor of 1.2, while activities in childhood and early adulthood were weighted by 1.0. The weighted counts across all life periods were summed to generate a total leisure score for each participant, reflecting both the diversity of activities and their persistence throughout the lifespan. Higher scores indicate greater engagement in diverse leisure activities over time, contributing to a higher estimated cognitive reserve. We then computed a single cognitive reserve composite score that included the scores for the three domains to produce an overall cognitive reserve index. This composite score reflects the cumulative contribution of educational attainment, occupational complexity and leisure activity engagement across the lifespan which provides a holistic measure of cognitive reserve without including foreign-accent exposure.

Occupational categories were weighted to reflect graded differences in cognitive demands associated with ISCO-based skill levels. This approach is in line with prior cognitive reserve research, demonstrating that occupations requiring greater cognitive flexibility, problem solving and decision-making contribute more strongly to reserve-related outcomes [21,22,34]. The modest scaling factors (1.2 for high complexity, 1.0 for medium, 0.8 for low) were chosen to preserve the contribution of employment duration while allowing occupational complexity to influence the composite proportionally. For education, all indicators (highest degree, years of schooling, and continued training) were standardized as z-scores and combined without additional weights, reflecting the view that each aspect contributes approximately equally to cognitive reserve. Similarly, leisure activities across the lifespan were counted by type and weighted modestly across adulthood periods (1.0 for childhood and early adulthood, 1.2 for adulthood and later adulthood) to reflect the increased relevance of sustained engagement in later life, while still preserving contributions from earlier experiences. These domain-specific decisions produced a composite CR score balancing duration, complexity and diversity of cognitively stimulating experiences. To evaluate whether findings depended on the weighting scheme used to construct the composite CR index, we recomputed the composite as an equal-weight mean of the three standardized domains (education, occupational complexity, leisure activity). The alternative composite was highly correlated with the original weighted version (r = 0.99). Re-estimating the mixed model with this equal-weight composite yielded an identical pattern of results (β = −0.03, *p* = 0.81) and confirmed that the null composite effect was not influenced by weighting assumptions.

### 2.4. Foreign Accent Rating Task

In the foreign accent rating task, participants listened to short speech recordings produced by non-native speakers of English and evaluated multiple perceptual dimensions of speech. The participants were allowed to listen to the stimulus as many times as they wished but were required to listen at least once before proceeding to the next item. They were asked to rate each recording on four perceptual dimensions using separate Likert-type scales: degree of foreign accent, comprehensibility, perceived intelligibility and listening effort. The degree of foreign accent was defined as the extent to which the speaker sounded non-native, independent of how easy the speech was to understand. Comprehensibility referred to the perceived ease or difficulty of understanding the speech, while perceived intelligibility reflected how much of the speech content participants felt they could accurately understand. Listening effort indexed the amount of mental effort required to follow the speech. All ratings were collected using eleven-point scales ranging from 0 to 10. For foreign accent, the scale ranged from 0 (“not accented at all”) to 10 (“very strongly accented”). For comprehensibility, the scale ranged from 0 (“very easy to understand”) to 10 (“very difficult to understand”). For perceived intelligibility, the scale ranged from 0 (“understood everything”) to 10 (“understood nothing”). For listening effort, the scale ranged from 0 (“no effort at all”) to 10 (“extreme effort”). For all scales except foreign-accent strength, higher numerical ratings indicate greater difficulty or effort; accordingly, positive effects of cognitive reserve on these outcomes reflect increases in perceived difficulty/effort, while negative effects indicate reductions in difficulty/effort. The speech stimuli for the Foreign Accent Rating task were obtained from the Speech Accent Archive [38]. All recordings consisted of speakers producing the standardized English passage “Please call Stella”. Only recordings that were free of hesitations, false starts, slips of the tongue or other speech disfluencies were selected. From each recording, only the first two lines of the passage were retained. Phrase-internal silent, breathing and hesitation pauses were removed while preserving the natural continuity of the speech. No speech segments were deleted or truncated. The resulting stimuli had durations ranging from 11 to 13 s. A total of 15 recordings were selected. The stimulus set was designed to ensure a controlled and diverse distribution with respect to speaker sex (male and female), age at recording, age of onset of English acquisition and geographic and linguistic origin. The final set included speakers from Africa, Europe, Asia and the Americas. English proficiency was assessed independently in a small-scale pilot norming study. In the norming phase, listeners evaluated the overall proficiency of the speakers and the ratings were used to classify recordings into proficiency levels. Although the age of onset of English acquisition was strongly associated with perceived proficiency, proficiency categorization was based on the norming results rather than solely on age-of-onset information. All stimuli were normalized for overall amplitude to ensure consistent playback volume across recordings. The order of the speech stimuli was randomized across participants. Audio stimuli were embedded directly in the survey and the participants were allowed to replay each recording as many times as needed, with a minimum of one complete playback required before proceeding.

### 2.5. Foreign Accent Exposure

To compute the foreign-accent exposure construct, we first considered multiple dimensions of exposure across the lifespan: current exposure, early adulthood (18–29 years), adulthood (30–49 years) and late adulthood (50–65 years). For each period, participants reported frequency of exposure (e.g., rarely, occasionally, frequently, very frequently), contexts of exposure (e.g., workplace, media, travel, friends, family) and level of interaction (mostly passive listening vs. occasional or regular direct interaction). We assigned numeric values to frequency (e.g., 0 = never, 1 = rarely, 2 = occasionally, 3 = frequently, 4 = very frequently) and interaction type (0 = mostly passive, 1 = occasional direct, 2 = regular direct) to quantify each period. For each participant, the frequency and interaction scores were combined across contexts within a period, producing a period-specific exposure score. Next, we aggregated scores across all life periods to compute a lifetime exposure index reflecting both breadth (number of contexts) and intensity (frequency × interaction). We further included self-reported measures of effortfulness in understanding foreign-accented speech, familiarity with foreign-accented speech, the number of different accents regularly encountered and self-rated confidence in evaluating accents as complementary indicators of exposure and experience. Finally, these indicators were standardized and combined to create a single composite foreign-accent exposure construct for use in the model, which allowed for capturing both quantitative exposure, subjective experience and familiarity with foreign-accented speech.

### 2.6. Statistical Modelling

Statistical analyses were conducted using mixed-effects models to account for the repeated-measures structure of the data with multiple recordings rated by each participant. The participant was included as a random intercept in all models. The ratings of foreign accent, comprehensibility, perceived intelligibility and listening effort were collected on Likert-type scales and were analyzed using cumulative link mixed models (CLMMs) with a logit link function (package *ordinal*), which are appropriate for ordinal outcome variables. Cognitive reserve (CR) was first entered as a composite score (CR_Composite_z) and included the combined contribution of occupational, educational and leisure components. This composite model served as the primary analysis. To examine the contribution of individual CR components, we first ran correlations among the three CR subcomponents (occupation, education, leisure). Although CR_leisure was excluded from component-level models due to high collinearity with occupational reserve (r = 0.75), we emphasize that leisure activities remain a core aspect of cognitive reserve and contribute meaningfully to the overall composite. The weighted approach described above ensured that leisure participation across the lifespan was incorporated in the composite score, capturing general attentional, social, and cognitive engagement. Sensitivity analyses confirmed that the overall pattern of results was unchanged when the composite was constructed with equal weighting across education, occupation, and leisure domains (r = 0.99 with the original weighted composite; β = −0.03, *p* = 0.81), indicating that the exclusion of leisure from component-level models did not bias the findings.

Reduced models, including CR_Occupation and CR_Education, were then fitted to assess their independent associations with each outcome. In all models, self-reported experience with foreign-accented speech was included as a covariate. Model estimates are reported with corresponding standard errors and significance values. Statistical analyses were performed in R (version 4.5.1).

## 3. Results

**Figure 1:**
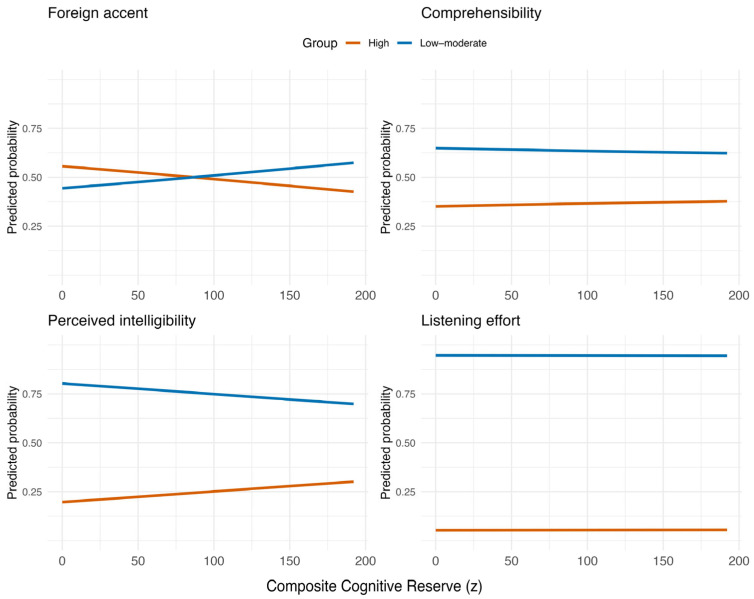
Predicted probabilities for the effect of composite cognitive reserve on the strength of the accent rating, comprehensibility, perceived intelligibility and listening effort.

Cognitive reserve (composite score) showed a marginal negative association with perceived foreign accent (β = −0.010, SE = 0.005, z = −1.92, *p* = 0.055), which should be interpreted cautiously given the small sample size and multiple outcome measures (Figure 1). Notably, more robust effects emerged when decomposing CR into occupational and educational components, which points to differential contributions of CR subdomains.

Self-reported experience with foreign-accented speech was not a significant predictor (β = 0.003, SE = 0.004, z = 0.69, *p* = 0.49). The random intercept variance for participants was 0.15, which points to little between-participant variability in overall accent ratings. For comprehensibility ratings, neither cognitive reserve (β = 0.002, SE = 0.007, z = 0.28, *p* = 0.78) nor foreign-accent experience (β = −0.002, SE = 0.005, z = −0.31, *p* = 0.76) significantly predicted ratings. Participant-level variability was moderate (random intercept variance = 0.33). Perceived intelligibility ratings were not significantly associated with cognitive reserve (β = −0.002, SE = 0.007, z = −0.29, *p* = 0.78) or foreign-accent experience (β = −0.001, SE = 0.006, z = −0.11, *p* = 0.91). The random intercept variance for the participant was 0.43. Listening effort ratings were also not significantly predicted by cognitive reserve (β = 0.004, SE = 0.006, z = 0.72, *p* = 0.47) or foreign-accent experience (β = −0.005, SE = 0.005, z = −1.05, *p* = 0.29). Participant-level variance was 0.28. All in all, cognitive reserve showed no reliable association with comprehensibility, perceived intelligibility, or listening effort and only a marginal association with perceived foreign accent. All results are presented in Table 1.

**Figure 2:**
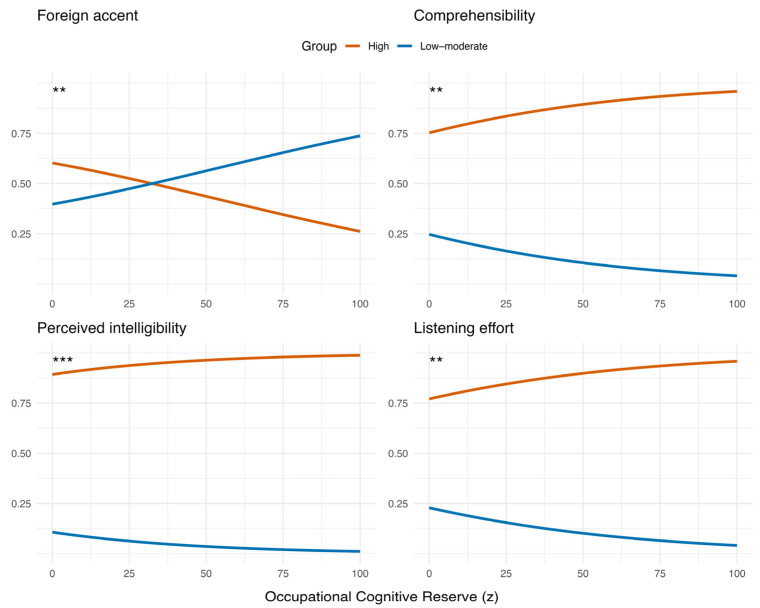
Predicted probabilities for the effect of occupational cognitive reserve on the strength of the accent rating, comprehensibility, perceived intelligibility and listening effort (p < 0.01 (**), p < 0.001 (***)).

**Figure 3:**
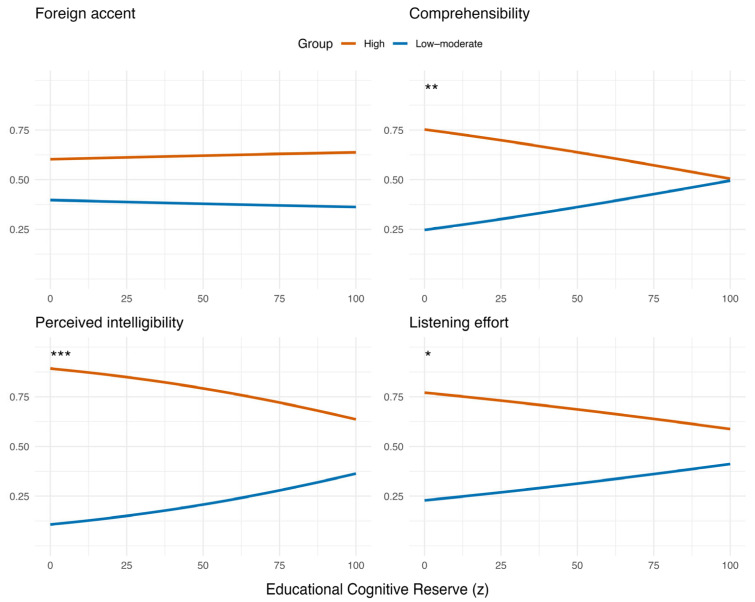
Predicted probabilities for the effect of educational cognitive reserve on the strength of the accent rating, comprehensibility, perceived intelligibility and listening effort (*p* < 0.05 (*), *p* < 0.01 (**), *p* < 0.001 (***)).

When cognitive reserve was decomposed into occupational (Figure 2) and educational components (Figure 3), clear and consistent effects emerged across outcomes. Occupational cognitive reserve was a significant predictor of comprehensibility (β = 0.020, *p* = 0.002), perceived intelligibility (β = 0.023, *p* < 0.001) and listening effort (β = 0.019, *p* = 0.003), indicating that listeners with higher occupational reserve were more likely to assign higher numeric ratings which correspond to greater problems with comprehensibility, perceived intelligibility ratings and effort ratings. In contrast, educational cognitive reserve showed significant effects in the opposite direction for the same outcomes (comprehensibility: β = −0.011, *p* = 0.002; perceived intelligibility: β = −0.016, *p* < 0.001; listening effort: β = −0.009, *p* = 0.014) indicating that higher educational reserve was associated with lower numeric ratings, i.e., better perceived comprehension and lower effort. Occupational cognitive reserve significantly predicted foreign accent ratings, with higher occupational reserve associated with lower perceived accent strength (β = −0.0166, *p* = 0.006). In contrast, educational reserve and self-reported foreign-accent experience did not significantly predict accent ratings across models. All results are presented in Table 2.

**Figure 4:**
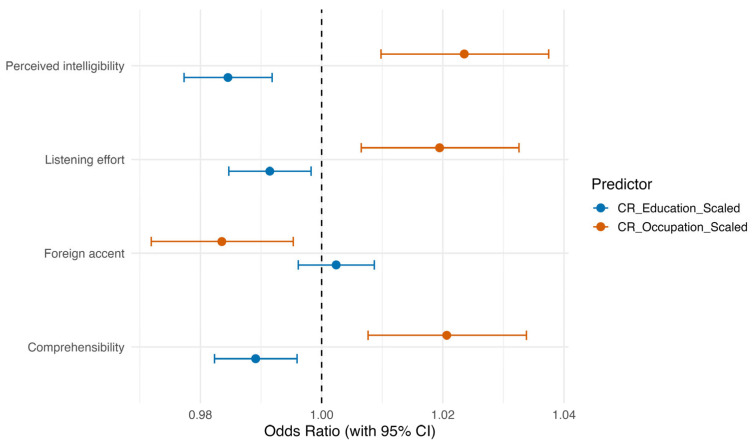
Odds ratios (ORs) and 95% confidence intervals (CIs) for the impact of occupational and educational cognitive reserve, and foreign-accent experience, on foreign accent ratings, comprehensibility, perceived intelligibility and listening effort. The dotted vertical line indicates Odds Ratio = 1 (null effect).

Figure 4 displays the odds ratios (OR) and 95% confidence intervals (CI) for each predictor. Across outcomes, occupational CR generally showed a slightly stronger effect than educational CR, whereas foreign-accent experience had minimal impact.

## 4. Discussion

The composite cognitive reserve results revealed only a marginal association with perceived accent strength and no reliable associations with comprehensibility, perceived intelligibility or listening effort. This finding suggests that overall cognitive reserve, when collapsed across its different proxies including education, occupation and leisure activities, does not strongly influence older adults’ explicit evaluations of foreign-accented speech. Thus, Hypothesis 1, which predicted a general association between overall cognitive reserve and perceptual evaluations of foreign-accented speech, was largely not supported. These findings align with previous work showing that older adults often achieve high levels of comprehension accuracy despite experiencing elevated processing demands [7,11]. Importantly, it indicates that subjective ratings of speech and particularly its perceived intelligibility, comprehensibility or listening effort, may not directly track the broad protective effects typically attributed to cognitive reserve in domains such as memory, executive function or global cognition. The marginal negative association between composite cognitive reserve and perceived accent strength suggests that listeners with higher reserve may be slightly less sensitive to accent salience or may normalize phonetic variability more readily. However, the effect was weak and did not extend to effort- or comprehension-related dimensions. This dissociation suggests that accent strength judgments may be less strongly related to the broad composite index of cognitive reserve than effort- or comprehension-related evaluations. While the overall reserve measure showed only a marginal association, subsequent analyses revealed that specific reserve components exert distinct effects, indicating that accent judgments are not uniformly resistant to reserve-related influences but may be sensitive to particular experiential factors.

A central contribution of the present study lies in decomposing cognitive reserve into its educational and occupational components. When examined separately, these components showed differential effects that were obscured in the composite analysis. Higher occupational reserve was associated with higher comprehensibility difficulty, lower perceived intelligibility and increased listening effort, whereas higher educational reserve predicted the opposite pattern, with lower difficulty and effort ratings but higher perceived intelligibility. Occupational reserve significantly predicted lower perceived accent strength, while educational reserve did not. These findings provide clear support for Hypothesis 2, which predicted that different components of cognitive reserve would differentially predict perceptual evaluations of foreign-accented speech. The beneficial effects of educational reserve may reflect more efficient comprehension monitoring or greater confidence in evaluative judgments rather than heightened perceptual sensitivity per se. Such effects of listener evaluative norms on accent and speech ratings have been documented in socio-phonetic research showing that experience and listener characteristics shape perceptual judgments independently of actual comprehension accuracy [39].

In contrast, the association between higher occupational reserve and more negative perceptual evaluations is less easily reconciled with traditional cognitive reserve accounts. Rather than reflecting poorer perceptual ability, this pattern may reflect differences in evaluative orientation or task engagement among individuals with more cognitively demanding occupational histories. Extensive professional experience may foster more analytically driven or differentiated judgments, leading to higher reported difficulty or effort despite comparable underlying comprehension. This interpretation is consistent with broader accounts of cognitive reserve, which emphasize that occupational complexity can enhance efficiency, attentional control, and flexible cognitive strategies in later life [33,34,35] and with research suggesting that listener experience and expertise shape perceptual and evaluative norms independently of objective performance [18,39].

To sum up, the opposing effects of occupational and educational reserve underscore the importance of treating cognitive reserve as a multidimensional construct. Aggregating heterogeneous life experiences into a single composite index may mask theoretically meaningful dissociations between different forms of enrichment and their distinct cognitive consequences.

Contrary to the initial hypothesis that composite cognitive reserve would be most strongly associated with effort-related dimensions, no such effect emerged for listening effort. However, educational reserve was a robust predictor of reduced effort, which leads to the conclusion that reserve mechanisms may primarily influence processing efficiency and evaluative confidence rather than perceptual categorization or evaluative judgments. This finding extends neurocognitive evidence indicating that older adults often recruit compensatory neural networks to maintain performance at the cost of increased effort [40]. Higher occupational reserve may mitigate this cost by enabling more efficient compensatory strategies. Notably, self-reported foreign-accent exposure did not significantly predict perceptual ratings in any model. While prior work has shown that repeated exposure can facilitate adaptation in younger listeners [5], the null effects observed here should be interpreted cautiously. Variability in lifetime exposure may have been restricted, and retrospective self-report measures are inherently limited, which may have reduced the sensitivity to detect any true association. Exposure was systematically assessed across multiple life periods and contexts, yet participants’ recollections, particularly for early and mid-adulthood experiences, may have been incomplete or imprecise. Such limitations in retrospective reporting could have reduced sensitivity to detect true associations. However, these results may also suggest that exposure alone may not reliably reduce perceived difficulty in older adults, and that broader cognitive and experiential factors, particularly those related to sustained educational or occupational engagement, may play a more central role in shaping subjective listening experiences later in life.

## 5. Limitations and Future Research Avenues

We acknowledge that this paper has limitations. Firstly, the sample included 30 participants, each providing multiple ratings. While cumulative link mixed models leverage repeated measures, the relatively small participant-level sample limits statistical power for detecting individual-difference effects, particularly when decomposing Cognitive Reserve (CR) into multiple components. Secondly, although our models accounted for participant-level random effects, item-level random effects were not included, despite the anticipation of some variability across speech stimuli. Thirdly, the leisure component of CR was excluded due to multicollinearity, which constrains the extent to which our analyses capture the multidimensional nature of cognitive reserve. A final limitation concerns the operationalization of intelligibility. Unlike classical transcription-based definitions (e.g., ref. [25]), our measure reflects listeners’ subjective perception of understanding. While this approach captures perceived communicative success, it does not permit conclusions about objective comprehension accuracy. Future research should aim to replicate these findings with larger samples to improve power for detecting individual-difference effects. Incorporating item-level random effects and including all dimensions of CR, particularly leisure activities, would allow a more comprehensive assessment of cognitive reserve. Additionally, comparing subjective and objective measures of intelligibility could clarify whether cognitive reserve predicts both perceived and actual speech comprehension. A further limitation concerns participants’ hearing abilities: although most reported no hearing impairments, even subtle, uncorrected hearing loss may have influenced perceptual ratings, particularly for speech intelligibility and listening effort.

## Figures and Tables

**Table 1:** The results of cumulative link mixed models for composite cognitive reserve and accent experience.

Outcome	Predictor	β (Log-Odds)	SE	z	*p*
Foreign accent	CR_Composite	−0.010	0.005	−1.92	0.055
	Accent experience	0.003	0.004	0.69	0.49
Comprehensibility	CR_Composite	0.002	0.007	0.28	0.78
	Accent experience	−0.002	0.005	−0.31	0.76
Perceived intelligibility	CR_Composite	−0.002	0.007	−0.29	0.78
	Accent experience	−0.001	0.006	−0.11	0.91
Listening effort	CR_Composite	0.004	0.006	0.72	0.47
	Accent experience	−0.005	0.005	−1.05	0.29

**Table 2:** The results of cumulative link mixed models for separate components of cognitive reserve and accent experience.

Outcome	Predictor	Estimate (β)	SE	z	*p*
Foreign accent	CR_Occupation	−0.0166	0.0061	−2.73	0.006
	CR_Education	0.0024	0.0032	0.75	0.453
	Accent experience	0.0026	0.0039	0.66	0.507
Comprehensibility	CR_Occupation	0.0204	0.0065	3.13	0.002
	CR_Education	−0.0109	0.0035	−3.12	0.002
	Accent experience	−0.0016	0.0041	−0.38	0.703
Perceived intelligibility	CR_Occupation	0.0233	0.0069	3.37	<0.001
	CR_Education	−0.0156	0.0038	−4.14	<0.001
	Accent experience	−0.0009	0.0042	−0.21	0.831
Listening effort	CR_Occupation	0.0193	0.0065	2.96	0.003
	CR_Education	−0.0086	0.0035	−2.46	0.014
	Accent experience	−0.0049	0.0041	−1.22	0.225

## Data Availability

All materials, raw data and analysis scripts for this study are publicly available at the Open Science Framework: https://osf.io/2hbsm/ (accessed on 23 January 2026).
